# Pulmonary Tuberculous Lesions: An Autopsy Study in Central Kerala, India

**DOI:** 10.7759/cureus.81632

**Published:** 2025-04-02

**Authors:** Hima A D, Lesitha S, Rajendra Prasad V K, Sheeju P A, Vasudevan P S, Suryakala R Nair, Shameem K Ummer Ali, Reena John, Sanjeev Nair

**Affiliations:** 1 Pathology, Government Medical College Idukki, Idukki, IND; 2 Pathology, Government Medical College Thrissur, Thrissur, IND; 3 Forensic Medicine, Government Medical College Kannur, Pariyaram, IND; 4 Forensic Medicine, Government Medical College Thrissur, Thrissur, IND; 5 Microbiology, Government Medical College Thrissur, Thrissur, IND; 6 Pulmonary Medicine, Government Medical College Thiruvanathapuram, Thiruvanathapuram, IND

**Keywords:** forensic autopsy, “granuloma”, kerala, prevalence study, tuberculosis

## Abstract

Introduction: Tuberculosis (TB) is a leading cause of mortality and morbidity in India. While India is moving towards ending TB, estimating the TB burden is still a challenge. This study aims to assess the prevalence of pathologically active TB in the lungs of deceased persons undergoing autopsy.

Methods: The study group consisted of all cases undergoing autopsy during the study period (May 2021-October 2022). Tissue bits from the apex of both lungs were collected during the autopsy. Gross pathological examination of both lungs and microscopy of tissue bits were done. Proportion and 95% confidence intervals were estimated for the prevalence of pathologically active TB. A chi-square test was applied to determine the factors associated with pathologically active TB.

Results: A total of 311 subjects were included in the study, of which 244 were male (78%), and the mean age was 51.32 (±15.9) years. The most frequent cause of autopsy was road traffic accidents, followed by death due to hanging. The proportion of pathologically active TB was estimated to be 2.89% (95%CI: 1.53%-5.41%). Nucleic Acid Amplification Test (NAAT) testing was done for a subset of 51 specimens, in which *Mycobacterium tuberculosis* was detected in only one case (1.96% (95%CI: 0.05%-10.45%)). Factors associated with the detection of lesions of TB were smoking, alcohol use, and prior history of TB.

## Introduction

Tuberculosis (TB) is an infectious disease caused by *Mycobacterium tuberculosis* (MTB). Globally, for a long time, TB was among the top 10 causes of death; however, it is now placed outside the top 10 for both the World Health Organisation (WHO) and Global Burden of Disease (GBD) estimates [1,2]. In addition to mortality, TB is also a leading cause of morbidity, being ranked ninth in the WHO list of top 10 causes of disability-adjusted life years (DALYs) for 2019 [3].

India is the top TB burden country in the world, with more than a quarter of the global incidence and global deaths due to TB occurring in India [4]. As per the Global TB Report 2023, an estimated 2.82 million people developed TB in India in 2022. As per the National TB Prevalence Survey of India, the highest estimate of prevalence is 534 per 100,000 population in Delhi, and the lowest is 115 per 100,000 in Kerala [5].

The End TB strategy is a highly ambitious program that aims at ending the global TB epidemic [6]. To achieve this, TB programs would have to diagnose and treat a very high proportion of cases. It is also necessary to have good estimates of the TB burden in the community. WHO has standardized TB prevalence study methodologies. However, these studies are time-consuming and costly [7]. The prevalence of TB infection in a community is estimated by doing tuberculin skin tests (TSTs) or Interferon Gamma Release Assays (IGRAs), which detect latent TB infection, and by conducting national population-based surveys or TB prevalence surveys [8].

It is estimated that Kerala has had a decline in the incidence of TB by 47% from 2015 to 2021 [9]. However, there is a lack of objective measures to show the decline. The state has not been able to take up state-specific prevalence studies to estimate the prevalence of TB so far, and the only data available is from the national TB prevalence study. In this context, it is hypothesized that the burden of pulmonary TB in Kerala could be estimated by the examination of lung samples obtained during autopsy, which is feasible and inexpensive.

## Materials and methods

This cross-sectional study was conducted in the Department of Forensic Medicine and the Department of Pathology in the Government Medical College, Thrissur, Kerala, India. All cases brought for autopsy during the study period (May 2021 to October 2022) were included in the study. Since the subjects were deceased, consent was taken from the relatives of the subjects. The study was cleared by the Institutional Ethics Committee of Medical College Thrissur (approval number: IEC/GMCTSR/108/2021 dated April 28, 2021).

The sample size was calculated as 289 based on the formula $n=\frac{Z^{2}\times pq}{d^{2}}$ with a correction for a finite population, where n = sample size, p= prevalence/proportion in previous study, q = 100-p, d = precision/allowable error, Z = Z score corresponding to desired level of confidence; p = 0.04, q = 100-p = 0.96, d= 0.008; n = 289.

Data collection

A structured questionnaire was used to collect basic data from the relative of the subject, including demographic information, co-morbidities, and habits. A whole-body autopsy was performed by the forensic surgeon. During autopsy, gross pathological examination of both lungs was done, findings were noted and recorded, and the organs were weighed and dissected. Two tissue bits from the apex of both lungs were taken for the study. One from each lung was preserved in 10% formalin and sent to the pathology department for histopathology examination. After routine processing and paraffin embedding, blocks were prepared and stained with hematoxylin & eosin. Ziehl-Nielsen staining was done to look for acid-fast bacillus (AFB) in all tissue blocks. They were examined microscopically, and findings recorded. The other tissue sample from both lungs was subjected to cartridge-based nucleic acid amplification testing (NAAT). The tissue was put in normal saline and transported to microbiology, cut into very small pieces with a sterile scalpel in a sterile Petri dish and the test was done and results recorded. Due to the NAAT machines being diverted for COVID-19 testing and the then-prevalent shortage of NAAT cartridges, NAAT could not be done in all samples and was limited to 51 samples. The presence of caseating granulomatous inflammation or caseation necrosis on microscopic examination or positive results in AFB or NAAT testing were considered to be diagnostic of TB.

Data analysis

Data was entered in Excel (Microsoft Corporation, Redmond, Washington, United States) and was analyzed using Epinfo 7.2.4.0 (Centers for Disease Control and Prevention, Atlanta, Georgia, United States). Categorical variables were summarized as percentages and quantitative variables as mean (± SD) or median (interquartile range (IQR)) as per the distribution. Proportion and 95% CI were estimated for the proportion of samples with a pathological diagnosis of TB as well as for the samples with NAAT positivity. A chi-square test was done to determine associations between the various factors and the pathological diagnosis of TB. Logistic regression was done to estimate the adjusted odds ratio (OR) (a priori decision of inclusion of those factors with p<0.1 in the logistic regression model).

## Results

**Figure 1:**
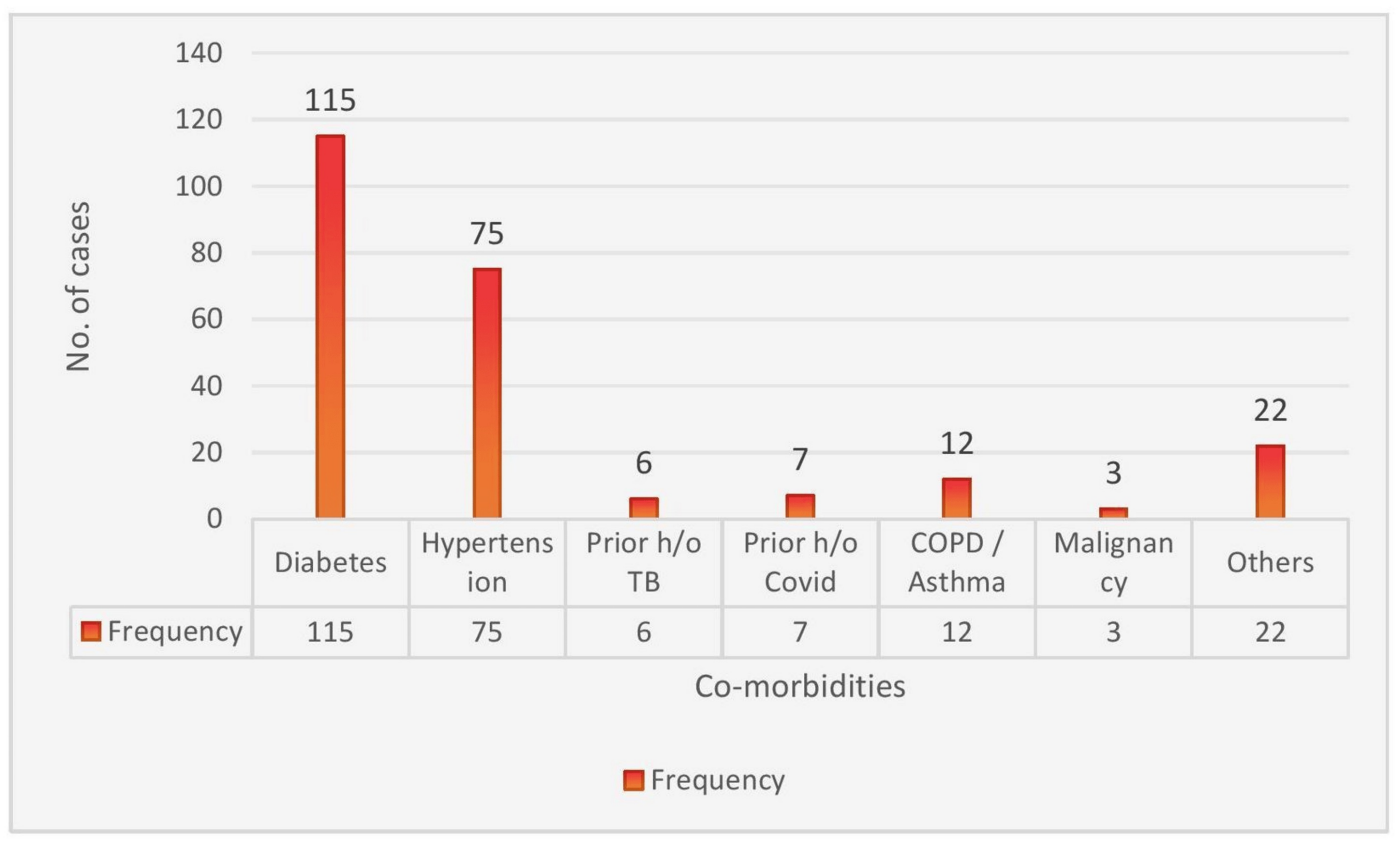
Distribution of co-morbidities (N=311) COPD: chronic obstructive pulmonary disease; TB: tuberculosis

The study group comprised 311 subjects brought for autopsy. Of these, 244 were male (78%) and 67 were female (22%). The mean age of the subjects was 51.32 years, with a standard deviation of 15.9 years. The median age was 53 years (IQR 40-63 years). The general characteristics of the subjects included in the study are given in Table 1 and co-morbidities in Figure 1. Diabetes was the most common co-morbidity seen in the study group, with 115 cases (37%), followed by hypertension in 75 cases (24%).

**Table 1:** General characteristics of the study group (N=311)

Variables	Group	Frequency	Percentage
Age	<50	135	43.41%
	>50	176	56.59%
Sex	Female	67	21.54%
	Male	244	78.46%
Personal history	Smoking	56	18.01%
	Alcohol abuse	42	13.50%

The most frequent cause of autopsy was road traffic accidents, and the next common cause was death due to hanging. The indications for autopsy in the subjects are given in Table 2.

**Table 2:** Distribution of cause of death (N=311) RTA: road traffic accident

Cause of death	Frequency	Percentage
Burns	8	2.57%
Drowning	14	4.50%
Hanging	80	25.72%
Myocardial infarction	8	2.57%
Occlusive coronary artery disease	35	11.25%
Poisoning	24	7.72%
Pulmonary thromboembolism	3	0.96%
RTA	104	33.44%
Others	35	11.25%

The most common pathological findings in the lungs of autopsy specimens were congestion and pulmonary edema, both on gross examination and in microscopy. The gross pathology and microscopy findings in the specimens are listed in Tables 3, 4. 

**Table 3:** Gross pathology findings of right and left lungs (N=311)

Finding	Left lung		Right Lung	
	Frequency	Percentage	Frequency	Percentage
Atelectasis	6	1.93%	6	1.93%
Bulla	3	0.96%	5	1.61%
Consolidation	23	7.40%	24	7.725
Cavity	2	0.64%	3	0.96%
Congestion	139	44.69%	136	43.73%
Necrosis	5	1.61%	6	1.93%
Oedema	117	37.62%	113	36.33%
Pus	14	4.50%	14	4.50%
Others	2	1.29%	4	1.29%

Those with extensive caseation necrosis and granuloma were diagnosed as TB. Nine cases (2.89%) were diagnosed as TB per the criteria (95%CI: 1.53%-5.41%) (Table 4).

**Table 4:** Distribution of microscopic findings of right and left lungs * Pathological diagnosis of tuberculosis

Microscopy findings	Right Lung		Left Lung	
	Frequency	Percentage	Frequency	Percentage
*Caseation necrosis	2	0.64%	1	0.32%
Collapse	5	1.61%	7	2.25%
Congestion	102	32.79%	93	29.90%
Congestion & emphysema	8	2.57%	7	2.25%
Chronic Venous Congestion	37	11.90%	37	11.90%
Diffuse alveolar damage	5	1.61%	4	1.29%
Fat emboli	0	0%	1	0.32%
Fibrosis	20	6.43%	28	9.00%
*Granuloma & caseation necrosis	7	2.25%	8	2.57%
Malignancy	3	0.96%	3	0.96%
Pneumonia	15	4.82%	15	4.82%
Pulmonary oedema	106	34.08%	104	33.44%
Pulmonary thrombi	1	0.32%	3	0.96%

At the start of the study, all the samples were expected to undergo NAAT testing. However, due to the COVID-19 pandemic-related shortages in test kits and NAAT machines being used for testing for COVID-19, NAAT testing in autopsy specimens was done only for 51 specimens, of which only one showed “MTB detected”. Thus, the proportion of microbiologically confirmed TB was estimated at 1.96% (95%CI: 0.05%-10.45%). Only one of the pathologically diagnosed TB cases underwent NAAT, and NAAT was positive in this case. AFB staining was done for all cases but was found positive only in two specimens (0.64%) (95%CI: 0.18%-2.31%). Both the positive AFB samples were cases that had pathological lesions of TB. Two of the nine pathologically diagnosed TB patients had a history of TB.

We also looked for the factors associated with a pathological diagnosis of TB. Smoking, alcohol use, and previous treatment for TB were found to be significantly associated with the pathological diagnosis of TB (P value < 0.05) (Table 5).

**Table 5:** Factors associated with a diagnosis of TB P value less than 0.05 was considered significant TB: tuberculosis

Factor	Group	Number	Number with diagnostic features of TB	Percentage	Odds ratio	95% CI		P value
						Lower	Upper	
Age (years)	>50	176	6	3.41%	0.64	0.16	2.62	0.39
	<50	135	3	2.22%				
Sex	Male	236	8	3.28%	2.24	0.21	18.27	0.38
	Female	67	1	1.49%				
Diabetes	Yes	115	5	4.35%	2.18	0.57	8.29	0.13
	No	192	4	2.04%				
Alcohol use	Yes	42	4	9.52%	5.56	1.43	21.61	0.02
	No	269	5	1.86%				
Smoking	Yes	56	5	8.93%	6.15	1.60	23.70	0.01
	No	255	4	1.57%				
Prior h/o TB	Yes	4	2	50%	21.29	3.33	136.12	0.01
	No	298	7	2.30%				

## Discussion

This study estimated the prevalence of pathologically diagnosed TB in autopsy specimens as 2.89% (95%CI: 1.53%-5.41%). The point estimate of 2.89% is much higher than the estimate of TB prevalence in Kerala in the National TB prevalence study, which estimated the prevalence of microbiologically confirmed pulmonary TB in Kerala at 0.115% (95%CI: 0.047%-0.184%) [5]. Thus, there is a huge difference in the estimated prevalence of TB for Kerala in the national prevalence study and the prevalence of pathologically diagnosed TB, the current estimate being almost 25 times the estimate from the prevalence study.

What could be the reasons for this difference in estimates? The TB prevalence study brings about the concept of prevalence to notification ratio, which indicates what proportion of cases may remain undiagnosed. Kerala, which fares well in other indicators, fares poorly in this indicator with a prevalence-to-notification ratio of 3.33 as opposed to the national average of 2.84. This indicates that a significant proportion of TB cases in Kerala may be remaining unnotified. Added to this are the current reports from all over the world on the occurrence of “sub-clinical TB” [10]. Multiple studies from various parts of the world have also shown a higher prevalence of TB detected on autopsy, as compared to clinical diagnosis or the prevalence of TB in that area [11-13]. A study from Ankara showed a prevalence of 1% [14], that from Switzerland showed 1.9% (with 44% undiagnosed cases) [15], and Japan showed 1% (with 74% undiagnosed cases) [16]. Studies from India have also shown a high prevalence of tuberculosis in autopsy specimens. A study from New Delhi showed the proportion of active tuberculosis lesions at 8.7% [17], from Chandigarh at 5% [18], 1.08% in Mangalore (diagnosis of TB known only in 12.1% of cases before death) [19], and 1.7% in Imphal [20]. The proportions seem to vary from state to state as the inclusion criteria, the diagnostic criteria, and the age groups are different.

The implications of the prevalence of lesions of pathologically active TB on autopsy specimens are not clear. However, India, as with the rest of the world, is attempting to End TB. One of the major targets of the End TB strategy is to reduce incidence by 90% and deaths by 95% by 2035 [6]. One of the major issues in this regard is the availability of data regarding trends in the TB burden. India had previously estimated the burden using annual risk of TB infection (ARTI) studies [21], which later became obsolete. Each of the methods of estimating the burden of TB has its peculiar problems [22]. The country has moved on to TB prevalence studies, but only one study has been done so far [5]. Follow-up studies are planned, but there are issues of cost and logistics that limit the widespread use of prevalence studies. Prevalence of active TB on autopsy studies can be easily done at academic institutes, the cost would not be high, and it would give an indirect estimate of the burden. This would also allow repeat estimates over fixed periods and give an idea of the trends in TB prevalence in that area.

The state of Kerala is supposed to be best placed to “eliminate TB” in India due to it having the lowest prevalence of TB [9]. The current notification of TB in Kerala is only 67/100,000 [23]. This often leads to complacency among administrators that TB is no longer a public health problem in Kerala. A recent study from one of the districts of the state showed an estimated age-standardized prevalence of TB Infection of 20.5% [24]. This finding and that of the current study showing the prevalence of pathologically active TB in autopsy specimens of 2.89% would indicate that there is still a long and arduous journey ahead for eliminating TB in Kerala. The implications of the prevalence of pathological lesions of TB during autopsy in comparison with TB prevalence in the community are not clear. However, the presence of such lesions in clinical specimens would have led to the diagnosis of TB and treatment. Hence, they have to be considered with due importance.

In the Kerala context, other probable reasons for a higher proportion in this study could be the higher age of subjects (median age of 53 years). There is evidence in Kerala about the higher proportion of TB in the higher age groups [9]. This study also looked at the risk factors for pathologically active TB and found that alcohol use, smoking, and a history of TB are significantly associated with the detection of TB. These risk factors have been included in the vulnerability mapping exercise of the TB elimination strategy of Kerala, and this study adds further justification for identifying such vulnerability. While diabetes was not a statistically significant risk factor in this study, the proportion of those with diabetes was more than twice the proportion of those without diabetes. In a state with a high prevalence of diabetes in TB [25], this is also a factor that needs to be properly addressed.

The authors would recommend screening for TB in autopsy specimens, with standardized methodology, including both pathological evaluations as well as evaluation with NAAT, as a multi-centre study should be taken up. The relationship between such an estimate and the prevalence of TB in community studies can be analysed. Multiple such studies over the years could give the country an indirect estimate of the trends in the TB burden in the country [26]. Such studies would be more feasible and much less costly than community-level TB-prevalence studies. Another recommendation would be that autopsy rooms should also be considered in the airborne infection control guidelines for the country, given the high prevalence of TB, to ensure the protection of healthcare workers in that setting.

The strength of this study is that a multidisciplinary team, including pathologists, microbiologists, forensic surgeons, and clinicians, was involved in the analysis. A large sample of 311 subjects undergoing autopsy was taken with sampling from both lungs. Data and sample collection were done by a single investigator to ensure consistency. The pathological reporting was done by two independent pathologists with more than 25 years of experience. The main limitation of this study is that microbiological testing with NAAT could be done only in a subset of the specimen due to a shortage in NAAT testing as a result of the COVID-19 pandemic.

## Conclusions

The prevalence of pathologically active tuberculosis in the lungs of autopsy subjects was 2.89% (95%CI: 1.53%-5.41%). Factors associated with the detection of lesions of TB were smoking, alcohol use, and prior history of TB. Autopsy studies can be taken up as a relatively easy and inexpensive methodology to asses the burden of the disease which is more feasible than community level TB prevalence studies. Furthermore, the findings from the study indicate the need for scaling up measures to look into hitherto unrecognized TB cases in the community.
